# Systematic Review and Meta-Analysis of Preoperative Antisepsis with Combination Chlorhexidine and Povidone-Iodine

**DOI:** 10.1055/s-0036-1587691

**Published:** 2016-08-10

**Authors:** Benjamin M. Davies, Hiren C. Patel

**Affiliations:** 1 Department of Neurosurgery, Greater Manchester Neuroscience Centre, Salford Royal Foundation Trust (SRFT), Salford, United Kingdom

**Keywords:** surgical site infection, surgical wound infection, antisepsis, chlorhexidine, povidone-iodine

## Abstract

**Importance** Effective preoperative antisepsis is recognized to prevent surgical site infection (SSI), although the definitive method is unclear. Many have compared chlorhexidine (CHG) with povidone-iodine (PVI), but there is emerging evidence for combination usage.

**Objective** To conduct a systematic review and meta-analysis to evaluate if combination skin preparation (1) reduces colonization at the operative site and (2) prevents SSI compared with single-agent use.

**Data Sources** A literature search of MEDLINE, Embase, and Cochrane Database of Clinical Trials was performed.

**Study Selection** Comparative, human trials considering the combination use of CHG and PVI, as preoperative antisepsis, to single-agent CHG or PVI use were included. Studies were excluded from meta-analysis if the use or absence of alcohol was inconsistent between study arms.

**Data Extraction and Synthesis** The study was performed using PRISMA (Preferred Reporting Items for Systematic Reviews and Meta-Analyses) guidelines.

**Main Outcomes and Measures** The primary outcome for meta-analysis was surgical site infection. The secondary outcome was colonization at the operative site.

**Results** Eighteen publications with a combination of CHG and PVI use were identified. Of these, 12/14 inferred promise for combination usage, including four trials eligible for meta-analysis. Only one trial reported SSI as its outcome. The remaining three considered bacterial colonization. Combination preparation had a pooled odds ratio for complete decolonization of 5.62 (95% confidence interval 3.2 to 9.7, *p* < 0.00001). There was no evidence of heterogeneity (Cochran's Q 2.1, 2 *df* , *p* = 0.35).

**Conclusions and Relevance** There is emerging, albeit low-quality, evidence in favor of combination CHG and PVI preoperative antisepsis. Further rigorous investigation is indicated.

Surgical site infection (SSI) is a significant cause of postoperative morbidity and mortality, increasing length of hospital stay and cost of care. 1 2 3 SSIs are the second most common cause of health care associated infection; U.S. studies have estimated a $45 billion annual cost. 4

Since Joseph Lister popularized the role of preoperative antisepsis in the 1800s, attempts to identify the optimal process and/or agent have resulted in several trials and much scientific tribulation. 5 Despite this effort, no absolute has emerged; a good example is the efforts to identify which of chlorhexidine (CHG) or povidone-iodine (PVI) is more effective. Several meta-analyses have been published, with no comprehensive conclusion in favor of one or the other. 6 7 8 9 10

CHG and PVI have different mechanisms of action and different spectrums of efficacy. Their simultaneous application was thought to form a less effective cocktail, although this belief has recently been challenged in vitro, with evidence in fact for a potential synergistic effect. 11 Regardless, sequential application would circumvent such concerns.

In clinical practice, therefore, no single chemical containing both CHG and PVI has been available. However, perhaps following the logic that “more is less” or covering all bases in the debate of CHG versus PVI, anecdotally and despite little scientific study of the method, many surgeons use a combination of CHG preparation and PVI preparation in sequence.

Our objectives therefore were to perform a systematic review and meta-analysis to consider the evidence for combination CHG and PVI antisepsis. Specifically, we aimed to study if combination skin preparation (1) reduces SSI compared with single-agent use and (2) reduces colonization at the operative site.

## Methods

### Systematic Review

MEDLINE (Ovid), Embase (Ovid), and Cochrane database of clinical trials (Ovid) were searched on April 1, 2015, using the search strategy (“Surgical site infection” OR “Surgical Wound Infection”) AND (“Chlorhexidine”) AND (“Iodine” OR “Iodine Compounds” OR “Povidone-Iodine” OR “Idophor”) adapted from Dumville et al. 6

A single reviewer screened resulting abstracts to select studies where a combination of CHG and PVI had been used. If unclear from the abstract alone, the full publication was sought. References from the short-listed abstracts were additionally screened for relevant studies. A second reviewer then rechecked this short list. Their quality of evidence was assessed by each reviewer independently and graded using the Oxford Levels of Evidence criteria. 12 Any disagreement was resolved by common discussion.

### Meta-Analysis

The primary outcome for the meta-analysis was SSI. The secondary outcome was decolonization at the operative site. For the meta-analysis, comparative, human trials comparing the use of combination CHG and PVI, as preoperative (i.e., just prior to incision) antisepsis, to single-agent CHG or PVI use were included. Studies were excluded if the use or absence of alcohol was inconsistent between arms of the study. Two reviewers assessed eligibility independently and any disagreement was resolved through discussion and mutual agreement. Assessment of bias was also conducted by each reviewer independently, using the criteria set out by Higgins et al. 13 This information was analyzed in combination with the GRADE profiling method, using GRADEpro (Evidence Prime, Hamilton, Ontario, Canada) to assess the overall quality of evidence relating to our primary and secondary outcomes. 14

Pooled analysis was conducted using Revman V5.3 (Cochrane Collaborative, Copenhagen, Denmark). Study heterogeneity was assessed using the Cochrane Q test, where *p* < 0.05 indicated significant heterogeneity and pooled odds ratios (ORs), with 95% confidence intervals (CIs), were calculated to assess overall effect.

## Results

**Fig. 1:**
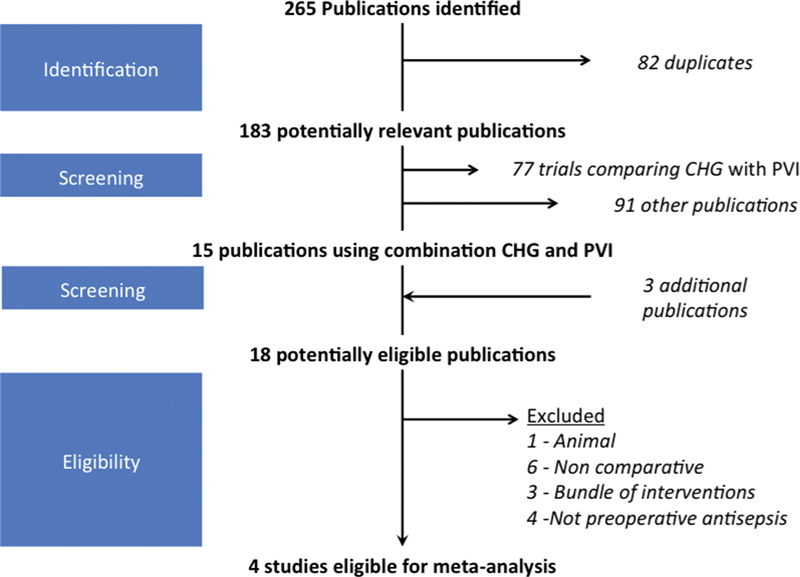
PRISMA (Preferred Reporting Items for Systematic Reviews and Meta-Analyses) flow diagram of search strategy. Abbreviations: CHG, chlorhexidine; PVI, povidone-iodine.

A PRISMA (Preferred Reporting Items for Systematic Reviews and Meta-Analyses) flow diagram for our search methodology and results is presented in Fig. 1 . This strategy identified 18 publications where a combination of CHG and PVI had been used ( Table 1 ). 1 11 15 16 17 18 19 20 21 22 23 24 25 26 27 28 29 30 Only four of these publications were eligible for the meta-analysis. 24 25 26 30

**Table 1:** Studies identified from the literature where combination CHG and PVI had been used

Year and reference	Publication study type	OEBM level	Population; objective	Method	Outcome measure; result	Comment
2013 15 a	RCT	N/A	*n* = 3,715; SSI after total knee arthroplasty	Standard practice (PVI or CHG skin prep) versus standard practice + preoperative CHG wipe 1 h before	Comparative data not available	Given standard practice included either PVI or CHG, the experimental arm would contain examples of patients with a combination PVI and CHG use; unfortunately these data were not available.
2014 16 b	Cohort study	4	*n* = 267; instrumented pediatric spinal surgery	Use of SSI bundle (drawn up based on expert consensus); included preparation with CHG and postoperative PVI	SSI incidence; preoperative bundle: 5.8%, postoperative bundle: 2.2%	Expert consensus was for combination CHG and PVI use.
1994 17 b	RCT	5	*n * = 473; pacemaker insertion	Antibiotics versus no antibiotics (patients had combination CHG/PVI skin preparation)	SSI incidence; no significant difference	Expert consensus was for combination CHG and PVI use.
1986 18 c	Cohort study	5	*n * = 12; hip arthroplasty	Standard practice (CHG bathing on morning of operation and day before) versus standard practice + preoperative PVI iodine rinse after second bath	Bacterial colonization no significant difference reported (individual data not shown)	Results suggest that a combination of PVI and CHG was not better than CHG alone.
2001 29 b	RCT	5	*n * = 50; foot or ankle surgery	Standard preparation (PVI + CHG) versus standard preparation + scrub with gauze-soaked swab	Bacterial colonization; standard preparation: 20.8%, additional scrub: 7.7%	Expert consensus was for combination CHG and PVI use.
2010 19 b	Cohort study	5	*n* = 126; patients with DM undergoing foot or ankle surgery	Ulcerated versus nonulcerated standard preparation; PVI + CHG	Bacterial colonization; no significant difference	Expert consensus was for combination CHG and PVI use.
2012 20 b	Letter	5	Plastic surgery	N/A	N/A	Expert opinion was for combination CHG and PVI use.
1971 30 b	Cohort study	5	*n * = 76; general surgery	CHG versus PVI + CHG	Bacterial colonization; PVI + CHG: 70% sterile, CHG: 36% sterile	Use of CHG + PVI was more effective at sterilization than CHG alone for decolonization.
2013 23 b	Quality improvement study	5	Spinal surgery	Bundle versus no bundle; bundle included CHG preparation, intraoperative irrigation with PVI	SSI incidence; preoperative bundle: 10.2%, postoperative bundle: 3.1%	Expert consensus was for combination CHG and PVI use.
2007 22 b	Quality improvement study	5	Pain medicine	Bundle versus no bundle; bundle required skin preparation with CHG + PVI	SSI incidence; nonsignificant reduction following introduction of bundle	Expert consensus was for combination CHG and PVI use.
2014 21 b	RCT	5	*n* = 1,874; arthroplasty or spinal fusion	Nasal decolonization; CHG + nasal mupirocin versus CHG + PVI	SSI incidence; CHG/mupirocin: 1.6%, CHG/PVI: 0.7%	Although not considering the operative site, this would suggest that a combination of CHG + PVI was better than CHG alone for decolonization of the nasal mucosa.
2002 27 b	RCT	5	*n * = 49; foot and ankle	Standard preparation (home CHG scrub + preoperative PVI) versus standard preparation + additional preoperative alcohol scrub	Bacterial colonization; CHG/PVI: 35%, CHG/PVI/alcohol: 57%	Data are not easily applied to our study outcomes, other than the fact a combination of CHG and PVI was clearly desired by the study authors.
2001 28 b	Cohort study	5	*n * = 1,038; neurosurgery	Head shaving versus no head shaving but CHG hair wash; standard preparation = CHG + PVI	SSI incidence; head shave: 1.2%, no head shave: 1.3%	Expert consensus was for combination CHG and PVI use.
2015 26 b	RCT	2	*n* = 1,394; obstetrics and gynecology	PVI versus CHG versus CHG + PVI	SSI incidence; overall no significant difference, but in obese women, combination significantly better	Combination was more effective in high-risk group.
1991 25 b	Cohort study	5	*n* = 242; cadaveric skin transplant harvest	PVI versus PVI + CHG	Bacterial colonization; PVI: 13.7%, CHG + PVI: 5.6%	Use of CHG + PVI was better than PVI alone for decolonization.
2009 24 b	Clinical trial	5	RCT *n* = 100; neurosurgery	CHG versus PVI + CHG	Bacterial colonization; CHG: 14%, CHG + PVI: 0%	Use of CHG + PVI was more effective at sterilization than CHG alone for decolonization.
2004 1 b	RCT	5	*n* = 140; ICU central line insertion	PVI versus CHG versus PVI + CHG	Bacterial colonization; PVI: 30.8%, CHG: 20.4%, CHG + PVI: 4.7%	Although not specifically for surgery, preprocedural antisepsis with a combination of CHG and PVI was more effective than either agent on its own for decolonization.
2010 11 b	Comparative study	5	In vitro/ex vivo (porcine)	PVI versus CHG versus PVI + CHG	Bacterial colonization; PVI, CHG, CHG + PVI: best	Although not a clinical study, this study provides evidence of the greater bactericidal effect of combination CHG and PVI.

Abbreviations: CHG, chlorhexidine; DM, diabetes mellitus; ICU, intensive care unit; N/A, not applicable; OEBM, Oxford Levels of Evidence-Based Medicine; PVI, povidone-iodine; RCT, randomized controlled trial; SSI, surgical site infection.

Note: The Oxford Evidence Based Medicine levels 1 to 5, where 1 is high quality evidence and 5 is low quality, have been used to consider the study in relation to our primary objective; does combination CHG and PVI reduce SSI?

a Evidence is unequivocal or unable to comment.

b Results support combination CHG and PVI.

c Results refute combination CHG and PVI.

Of the excluded but short-listed studies, 12/14 offered some indication of a role for combination CHG and PVI skin preparation, including 3 studies detailing the implementation of a bundle of care requiring combination skin preparation, 16 22 23 1 letter in favor of its use, 20 5 clinical trials where combination skin preparation was standard practice in both arms, 17 19 27 28 29 1 trial in favor of combination CHG and PVI for preoperative nasal decolonisation, 21 1 trial in favor for central line insertion, 1 and 1 basic science study. 11 Only 1 study suggested the combination provided no additional benefit. 18 These publications were considered level 5 evidence.

### Primary Objective: Does Combination Skin Preparation Reduce Surgical Site Infection Rates?

Only 1 of the 4 eligible studies reported SSI as the outcome. This study was a conference abstract of a randomized controlled trial (RCT) of 1,404 women undergoing cesarean section to preoperative antisepsis with PVI, CHG, or combination CHG and PVI by Ngai et al. 26 During analysis, the article was subsequently published in full. 31 The study identified SSI incidences of 4.6% PVI, 4.5% CHG, and 3.9% CHG and PVI. This difference was not significant. However, on performing subgroup analysis of class III obese women, a combination of CHG and PVI was demonstrated to reduce SSI in a multivariate model (OR 0.17, CI 0.04 to 0.77).

### Secondary Objective: Does Combination Skin Preparation Reduce Bacterial Colonization at the Operative Site?

**Fig. 2:**
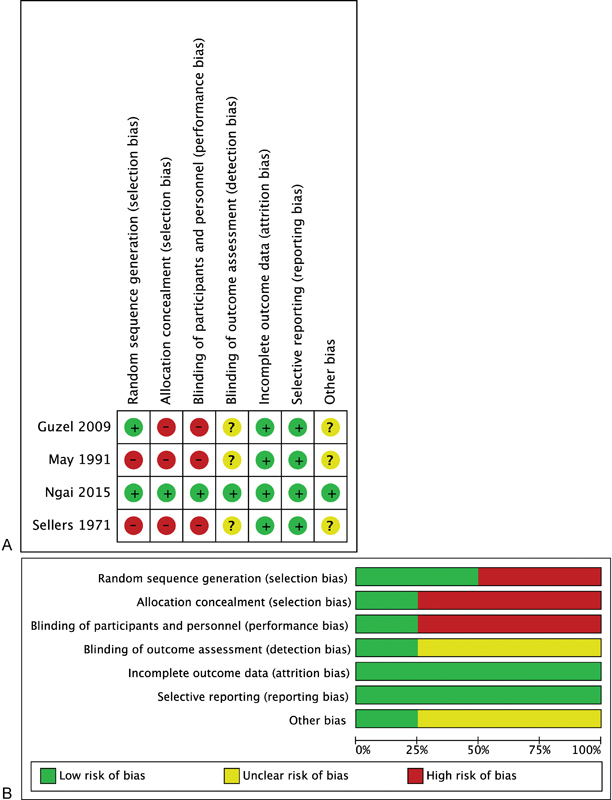
Risk of bias summary (A) and graph (B) for studies eligible for meta-analysis.

The remaining three studies reported bacterial colonization as their outcome. 24 25 30 The quality of evidence had a moderate to high risk of bias, given the methodologies were susceptible to selection, performance, and detection bias ( Fig. 2 ). Specific discrepancies included the tissue choice (grafted skin tissue, 25 regular skin, 24 and the umbilicus 30 ), the application (CHG/alcohol/PVI, 25 CHG/PVI/PVI, 24 and PVI-soaked sponge for 1 hour/CHG with alcohol 30 ), and the control preparation ( Table 2 ).

**Table 2:** Comparison of the study design and methodology for those studies included in a meta-analysis of bacterial decolonization

	Sellers and Newman 30	May et al 25	Guzel et al 24
Tissue type	Human umbilicus in abdominal surgery	Harvested human skin grafts	Neurosurgical operating site (cranial and spinal)
Sample	*n* = 105; patients were treated as separate samples	*n* = 342; grafts were taken from multiple sites, yielding 3,263 samples	*n* = 100; patients were treated as separate samples; 50 cranial and 50 spinal patients
Control arm	*n* = 70	*n* = 294 (2,940 samples)	N/A
Combination arm	*n* = 35	*n* = 48 (323 samples)	N/A
Application method (including timing if specified)	PVI-soaked sponge for 1 h, CHG with alcohol	CHG; alcohol; PVI	CHG 3 min; PVI 30 s; PVI 30 s
Control antisepsis	CHG with alcohol	PVI with alcohol; PVI	Samples for culture counts were taken in between cleanings; therefore counts after CHG only are compared with CHG and PVI

Abbreviations: CHG, chlorhexidine; DM, diabetes mellitus; N/A, not applicable; PVI, povidone-iodine.

In addition, it was often unclear as to whether adequate precautions had been taken to neutralize the samples during bacterial counting.

The outcomes of these studies also differed, but they all reported the proportion of individual patients yielding no growth. Overall, therefore the result for 547 patients could be pooled for analysis.

**Fig. 3:**
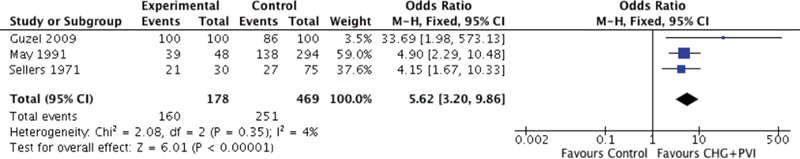
Forrest plot showing the pooled effect of combination CHG and PVI on complete bacterial decolonization. Abbreviations: CHG, chlorhexidine; PVI, povidone-iodine.

Complete decolonization rates were greatest for combination CHG and PVI (90%), compared with CHG (65%) or PVI (47%) alone, and yielded a pooled OR for complete decolonization of 5.62 (95% CI 3.2 to 9.7, *p* < 0.00001) in favor of combination CHG and PVI skin preparation ( Fig. 3 ). There was no evidence of heterogeneity (Cochran's Q 2.1, 2 *df* , *p* = 0.35).

## Discussion

At present, only a single high-quality RCT has considered the effect of a combination of CHG and PVI with some promise. The evidence for improved bacterial decolonization with a combination of PVI and CHG is perhaps firmer.

Although a reduction in SSI is a logical result of greater bacterial decolonization, studies have reported contradicting examples. 32 33 The relative infrequency of SSI has led to the acceptance of surrogate markers such as skin cultures, 34 but their association with SSI is not well validated. 7 As a multifactorial problem, it is likely that the effect was hidden, but it poses a hurdle for future investigation. 34

This difficulty can be seen in the RCT by Ngai et al. 31 During their study, they found an unexpected low incidence of SSI, which resulted in an underpowered study. Post hoc analysis suggested a sample size of 3,000 would have been required to detect a significant difference. 31 Although such a large-scale study would be unusual in surgery, the requirement for improved methods of combatting SSI is clear. SSI significantly contributes to postoperative morbidity and mortality, and with worsening antibiotic resistance, it could become more pertinent. Powering to detect a 0.5% improvement therefore has major implications, when its extrapolation across different fields of surgery all around the globe is considered.

An additional question to be answered is whether the efficacy of combination skin preparation draws on the intrinsic requirement for the skin to be cleaned twice rather than the agent itself. There is some evidence to suggest this factor is not significant: May et al controlled for this eventuality when comparing PVI with CHG and PVI, 25 and O'Shaughnessy et al and Langgartner et al found the decolonization effect of CHG is not time-dependent. 1 35 However, Morrison et al, using iodine and alcohol, found double preparation more effective. 36

Placing our findings within the existing body of literature is difficult, given its paucity. The pooled analysis identified that PVI was less effective than CHG, which reassuringly is in keeping with consensus from the literature. 6 8 Additionally, the greater efficacy of combination CHG and PVI mirrors findings from other types of antisepsis and its incorporation into successful bundles of perioperative care. 1 16 22 23

On the background of our findings from this systematic review, we have conducted a retrospective assessment of our neurosurgical surveillance data from a single center. 37 In a multivariate model, we identified a greater than fivefold benefit for combination CHG and PVI (OR 0.12, CI 0.02 to 0.63).

Mechanistically, there is good reason to believe combination CHG and PVI would be of benefit. First, although both have a broad spectrum of antibacterial activity, PVI can also target viruses, fungi, and bacterial spores, and CHG can target yeast. Second, the action of PVI is intracellular, and therefore the action of CHG, which disrupts cell membranes, would theoretically augment its potency. And finally, PVI has a more immediate action than CHG, which is delayed. 11 38

## Conclusion

Further advances in the battle to prevent SSI are required as its significant impact is well recognized. Bacterial decolonization at the operative site is more effective when the combination is used. Although there is presently no level 1 evidence demonstrating a definitive effect of combination CHG and PVI for reducing SSI, building on promising mechanism-based reasoning, basic science data, and its incorporation into successful bundles of care, the use of combination CHG and PVI has shown promise in a large RCT. Further study is warranted.
